# Study on Three *Sarcocapnos* Species as Potential Sources of Bioactive Compounds: Relation between Phenolic Content and Bioactivity by Multivariate Analysis

**DOI:** 10.1155/2020/8885169

**Published:** 2020-07-11

**Authors:** María del Pilar Fernández-Poyatos, Gökhan Zengin, Carlos Salazar-Mendías, Antonio Ruiz-Medina, Kouadio Ibrahime Sinan, Eulogio J. Llorent-Martínez

**Affiliations:** ^1^Department of Physical and Analytical Chemistry, Faculty of Experimental Sciences, University of Jaén, Campus Las Lagunillas, Jaén E-23071, Spain; ^2^Department of Biology, Science Faculty, Selcuk University, Campus, Konya, Turkey; ^3^Department of Animal Biology Plant Biology and Ecology, Faculty of Experimental Sciences, University of Jaén, Campus Las Lagunillas, Jaén E-23071, Spain

## Abstract

In this work, we report the phenolic composition and bioactivity of the aerial parts of three species of *Sarcocapnos* (*S. enneaphylla*, *S. pulcherrima*, and *S. saetabensis*) to study their potential as sources of bioactive compounds to revalorize them and contribute to the conservation of these plant species. Samples were collected in different locations in the province of Jaén (southeast of Spain), and qualitative and quantitative analyses of phenolic compounds were performed by high-performance liquid chromatography with diode array and mass spectrometry detection. *S. enneaphylla* presented the highest concentration of phenolic compounds (58 mg/g DE). The most abundant compound in *S. enneaphylla* and *S. saetabensis* was rutin (35 mg/g DE and 11.7 mg/g DE, respectively), whereas isorhamnetin-O-rutinoside was dominant in *S. pulcherrima* (11.5 mg/g DE). Several assays were performed to evaluate the potential bioactivity of the three species of *Sarcocapnos*. These assays included antioxidant and radical scavenging (ABTS and DPPH), reducing power (CUPRAC and FRAP), phosphomolybdenum and metal chelating, and enzyme inhibitory activity (acetylcholinesterase, amylase, butyrylcholinesterase, glucosidase, and tyrosinase). In general, all methanolic extracts presented the highest phenolic and flavonoid contents, as well as the highest radical scavenging, antioxidant, and enzyme inhibitory properties. This relationship between phenolics and bioactivity was confirmed by multivariate analysis.

## 1. Introduction

Plants are valuable sources of bioactive compounds of outstanding interest for the pharmaceutical industry. Due to the side effects of synthetic drugs, the interest in natural medicinal plants has increased in the last decade. Phenolic compounds are among the most important phytochemicals of plants due to their important health benefits [1]. The use of natural products is not only exclusive for human health but also for applications in livestock and aquaculture production systems [2]. It is thus important to characterize the composition and bioactivity of phytochemicals in lesser-known plants.

*Sarcocapnos* DC. (subfam. *Fumarioideae*, fam. Papaveraceae) is a plant genus endemic to the Western Mediterranean subregion (southwest Europe and northwest Africa) which has been scarcely investigated so far. Papaveraceae plants have been widely used due to their medicinal properties. Among them, several *Sarcocapnos* species have been consumed as infusions for the treatment of several illnesses. *Sarcocapnos* genus is composed of ten taxa: seven species and three subspecies [3, 4]. Its main diversification center lies in the Iberian Peninsula, from which eight out of the ten accepted taxa are endemic [5]. Except for the northwest African endemism *S. crassifolia* (Desf.) DC., the rest of the six species are present in Spain. Some of them show a narrow distribution area circumscribed to the Baetic mountains of Southern Iberian Peninsula (Spain), mainly distributed in the area of eastern Andalusia [6].

All *Sarcocapnos* species are present in overhanging cliffs, mainly over carbonated substrata. Most of them are endangered species [7], usually threatened by their own biological and demographic features, natural processes (droughts and rock collapse), global climate change, and at a lesser extent, by human activities such as quarrying or climbing. Only a few of them are currently protected by law at a national or regional level. Therefore, studies concerning their chemical composition and bioactivity are important to revalorize and to protect these plant species.

The genus *Sarcocapnos* has proved to be a rich source of isoquinoline alkaloids, most of them with a cularine skeleton [8–10]. However, to our best knowledge, the phenolic composition of *Sarcocapnos* species has not been reported to date. Also, there are no studies regarding the bioactivity of these plant species. We have therefore studied the phenolic composition and bioactivity of three species (all of them suffruticose chasmophytes) that coexist in the province of Jaén (southeast of Spain): *S. enneaphylla*, *S. pulcherrima*, and *S. saetabensis*.

*S. enneaphylla* (L.) DC. has the widest distribution area of the genus, from Southern France to Northern Africa, with a great number of populations throughout Spain [11]. Its flowers and leaves show the smallest dimensions of the genus. *S. pulcherrima* Morales and Romero García is an endemic species to southeastern Spain, with isolated populations in Eastern Andalusia [12]. And finally, *S. saetabensis* Mateo and Figuerola species seems to have a hybrid origin from *S. enneaphylla* and *S. pulcherrima* [13]. It is an endemic species to Eastern Spain from Catalonia to eastern Andalusia. The alkaloid compositions of the three *Sarcocapnos* species have been previously reported [8, 9, 14].

Considering the scarce data concerning the mentioned species, the first goal of this work is to detail the composition of the phenolic content of aerial parts of these *Sarcocapnos* species as well as their antioxidant activity and enzyme inhibitory properties against cholinesterase, amylase, glucosidase, and tyrosinase. The final aim is to develop novel pharmaceutical and food products based on the extracts of these plants (or specific isolated bioactive compounds), therefore valorizing these species as a source of bioactive compounds and hence contributing to their conservation.

## 2. Materials and Methods

All chemicals and reagents are given in File S1 of Supplementary Materials.

### 2.1. Plant Material and Sample Preparation

**Figure 1:**
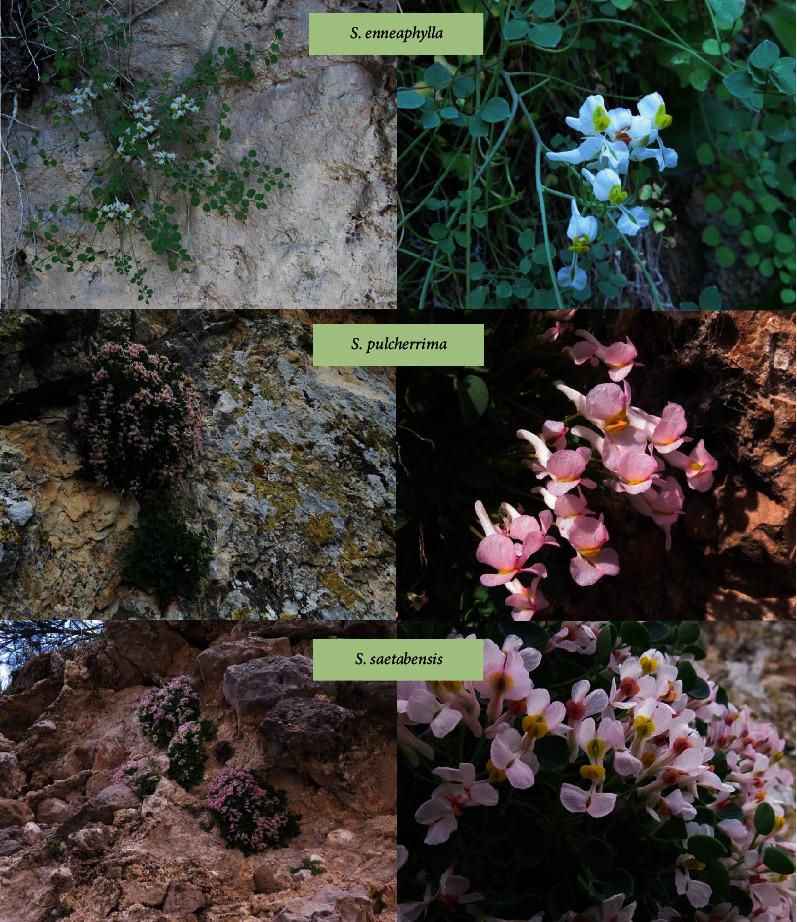
Photographs of *S. enneaphylla*, *S. pulcherrima*, and *S. saetabensis*.

*Sarcocapnos* leaves were collected by hand from different locations (two locations per plant species) in the province of Jaén (Southeast of Spain) as shown in Table 1. Plant material of each location is a pool of the aerial parts collected from approximately five different plants during the same day.  Figure 1 shows the photographs of each *Sarcocapnos* species in their collection site. The taxonomical classification was confirmed by botanist Dr. Carlos Salazar-Mendías, and voucher numbers are also given in Table 1.

Extractions were carried out in two different media: methanol (MeOH; HPLC grade; Sigma-Aldrich) and water (Milli-Q Waters purification system; Millipore; Milford, MA, USA). Due to the small amount of plant material (most *Sarcocapnos* species are endangered), we selected MeOH and water, the most common solvents for the extraction of phenolic compounds. For both extractions, leaves were lyophilized (ModulyoD/23, Thermo Savant; Waltham, MA, USA) and crushed with a grinder.

Methanol extractions were performed as follows: 2.5 g of dry material was extracted with 50 mL MeOH in an ultrasonic liquid processor (Qsonica Sonicators; Newton, CT, USA) with a power of 55 W and a frequency of 20 kHz, for 10 min (using 50% power) at room temperature. Extractions were done in triplicate. After sonication, solutions were filtered through Whatman No.1 filters. The solvent was evaporated under reduced pressure in a Hei-Vap Precision rotary evaporator (Heidolf; Schwabach, Germany) at 40°C. Dried extracts (DEs) were stored at –20°C until analysis.

On the other hand, the extractions with water were carried out in the following way: 2.5 g of dry material was extracted with 150 mL H_2_O at 100°C in a hot plate (C-MAG HS7, IKA; Staufen, Germany) for 30 min. Extractions were done in triplicate. After that, solutions were filtered in a vacuum pump (Vacuubrand; Wertheim, Germany) through Whatman No. 1 filters. Finally, the solvent was evaporated under reduced pressure, and the dried extracts were stored at –20°C until analysis.

### 2.2. HPLC Analysis of the Phenolic Compounds

The extracts were analyzed with high-performance liquid chromatography with diode-array and mass spectrometry detection (HPLC-DAD-MS^n^), operating in both negative and positive ion modes. Instrumentation and detailed conditions are given in File S2 of Supplementary Materials.

Five milligrams of DE (MeOH) was redissolved in 1 mL of MeOH, and 5 mg of DE (H_2_O) was redissolved in 1 mL of MeOH : H_2_O (10 : 90; v:v). After filtration through 0.45 *μ*m nylon membrane filters for methanolic extracts and 0.45 *μ*m PVDF membrane filters for aqueous extracts, 10 *μ*L of sample were injected.

Individual sock solutions of caffeic acid, neochlorogenic acid, coumaric acid, ferulic acid, hydroxytyrosol, sinapic acid, quercetin, kaempferol, and rutin were prepared in MeOH. We prepared calibration curves for caffeic acid, neochlorogenic acid, coumaric acid, ferulic acid, hydroxytyrosol, sinapic acid, quercetin, and rutin at concentrations 0.5–100 *μ*g mL^−1^ in MeOH. Chromatograms were recorded at 280 nm for hydroxytyrosol; 320 nm for caffeic acid, neochlorogenic acid, coumaric acid, ferulic acid, and sinapic acid; and 350 nm for quercetin and rutin. Peak area (at the corresponding wavelength) was plotted vs analyte concentration to construct the calibration graphs. Repeatability (*n* = 9) and intermediate precision (*n* = 9, 3 consecutive days) were lower than 3 and 8%, respectively. The robustness of the method was assessed by measuring signals at ±2 nm of the optimum wavelength and by modifying the percentage of mobile phases (2% variation with respect to optimum conditions), observing variations lower than 5% in all cases.

### 2.3. Assays for Total Phenolic and Flavonoid Contents

Total phenolic content (TPC) and total flavonoid content (TFC) were measured by spectrophotometric assays [15]. The obtained results were reported as standard equivalents of gallic acid and rutin for phenolics and flavonoids, respectively. Details for the protocols are provided in File S3 of Supplementary Materials.

### 2.4. Determination of Antioxidant and Enzyme Inhibitory Effects

Regarding the antioxidant activity of *Sarcocapnos* extracts, different spectrophotometric assays were performed: ferrous ion chelating, phosphomolybdenum, FRAP, ABTS, CUPRAC, and DPPH [15]. Results are given as standard compounds equivalents of Trolox and EDTA. Details are given in File S4 of Supplementary Materials.

The in vitro enzyme inhibitory effects of *Sarcocapnos* extracts were evaluated on five enzymes: *α*-amylase, *α*-glucosidase, acetyl- and butyryl-cholinesterases, and tyrosinase. The enzyme inhibitory actions were assessed as kojic acid equivalents (KAE) for tyrosinase, galantamine equivalents (GALAE) for acetylcholinesterase (AChE) and butyrylcholinesterase (BChE), and acarbose equivalents (ACAE) for *α*-amylase and *α*-glucosidase. Assays were performed as previously reported [15, 16], and the details for the protocols are provided in File S4 of Supplementary Materials.

### 2.5. Statistical Analysis

The different *Sarcocapnos* extracts were analyzed in triplicate (there were 3 independent extracts for each plant species; Section 2.1), and the values were given as mean ± SD. Firstly, the data were submitted to a descriptive analysis (ANOVA one-way) followed by Tukey's test, using XLSTAT v.2018 (Addinsoft Inc) statistical software. A significance level of 5% was set for all analyses. Unsupervised principal component analysis (PCA) was performed in order to investigate similarities/differences between different samples and to identify the factors responsible for the distinguishing between that samples. Prior to principal component analysis, data were prepreprocessed (autoscaling). Afterwards, Hierarchical Cluster Analysis (HCA) was applied on the results of PCA to bring out the different clusters. To this end, the Euclidean similarity measure and complete linkage were chosen. Finally, Pearson's correlation was carried out to assess the relationship between total bioactive compounds and biological activities. All statistics were done under R v.3.6.1 software.

## 3. Results and Discussion

### 3.1. HPLC-MS Analysis

Mass spectrometry was used for compounds characterization. A typical chromatogram is shown in Figure S1 of Supplementary Materials. We identified or tentatively characterized 37 compounds, of which more than 30% corresponded to flavonoids and more than 40% were phenolic acids. The characterization of the compounds was performed by using information available in scientific literature and the use of analytical standards. Compounds were numbered according to their order of elution (Table 2), maintaining the same numeration in all samples.

#### 3.1.1. Flavonoids

Compounds **12**, **21**, **27**, and **30** were identified as quercetin-O-glycosides. All of them displayed neutral losses of 146, 162, and 308 Da, which corresponded to deoxyhexoside, hexoside, and rutinoside moieties. The presence of quercetin was confirmed by the fragment ions at *m*/*z* 301, 179, and 151 (comparison with an analytical standard). The identity of rutin (compound **27** was confirmed by comparison with an analytical standard).

Compounds **17**, **25**, **34**, **35**, **36**, and **38** were isorhamnetin-O-glycosides. They were also characterized by the neutral losses previously mentioned, as well as the loss of acetylhexoside (204 Da) in compound **38**. In all of them, the aglycone isorhamnetin was observed at *m*/*z* 315 (typical fragment at *m*/*z* 300; comparison with an analytical standard).

Compounds **31** and **33** were putatively characterized as kaempferol-O-rutinoside isomers due to the loss of rutinoside (308 Da) and the presence of the aglycone kaempferol at *m*/*z* 285 (the aglycone was identified as kaempferol due to the absence of MS^3^ fragment ions at *m*/*z* 243 and 241, which would be indicative of luteolin; analytical standards of both kaempferol and luteolin were analyzed).

#### 3.1.2. Phenolic Acids

Compounds **3**, **11**, **16**, **20**, **22**, and **24** were characterized as feruloylquinic acid isomers. Compounds **3** and **11** displayed deprotonated molecular ions at *m*/*z* 367 with MS^2^ and MS^3^ base peaks at *m*/*z* 193 and 134, respectively; they were identified as 3-feruloylquinic acid isomers [17]. Compounds **16** and **20** presented [M − H]^−^ ion at *m*/*z* 367, with MS^2^ and MS^3^ base peaks at *m*/*z* 173 and 111, respectively; they were characterized as 4-feruloylquinic acid isomers [17]. Compounds **22** and **24**, with [M − H]^−^ ion at *m*/*z* 367 and MS^2^ base peak at *m*/*z* 191, were characterized as 5-feruloylquinic acid isomers [17].

Compound **8** with [M − H]^−^ ion at *m*/*z* 353 presented fragment ions at *m*/*z* 191 and 179, which corresponded to neochlorogenic acid (identified by comparison with an analytical standard).

Compounds **9** and **10** were characterized as 3-p-coumaroylquinic acid isomers based on the [M − H]^−^ ions at *m*/*z* 337, MS^2^ base peak at *m*/*z* 163, and comparison of their fragmentation pattern with bibliographic data [17].

Compound **14** was characterized as ferulic acid glucuronide; it exhibited deprotonated molecular ion at *m*/*z* 369 and suffered the neutral loss of 176 Da (glucuronide) to yield fragment ions at *m*/*z* 193 and 134, typical of ferulic acid. Compound **28** corresponded to ferulic acid (identified by comparison with an analytical standard).

Compound **19**, [M − H]^−^ at *m*/*z* 295, had major fragments at *m*/*z* 179 and 135, which indicated the presence of caffeic acid; it was characterized as caffeic acid cinnamyl ester [18].

Compound **23** presented [M − H + HCOOH]^−^ at *m*/*z* 453 and was tentatively identified as coumaric acid-O-hexoside derivative (formate adduct) due to the neutral loss of hexoside (325 ⟶ 163) and the presence of coumaric acid (fragmentation 163 ⟶ 119). Compound **26** was identified as coumaric acid by comparison with an analytical standard. Compound **29** was tentatively characterized as a coumaric acid derivative.

Compound **32** displayed deprotonated molecular ion at *m*/*z* 683, with fragments at *m*/*z* 223 and *m*/*z* 205, typical of the sinapic acid fragmentation, so this compound was tentatively identified as sinapic acid derivative.

#### 3.1.3. Other Compounds

Compound **1** was identified as a disaccharide (HCl adduct) formed by two hexosides (*m*/*z* 341). The fragment ions at *m*/*z* 179 and 161 are typical from hexoside moieties.

Compounds **2** and **4** were characterized as isocitric acid and citric acid, respectively. Both showed [M − H]^−^ ion at *m*/*z* 191, but citric acid exhibited two fragment ions at *m*/*z* 111 (base peak) and *m*/*z* 173, while isocitric acid showed fragment ions at *m*/*z* 173 (base peak), *m*/*z* 155, and *m*/*z* 111 [19].

Compound **6** presented [M − H]^−^ ion at *m*/*z* 315 and suffered the neutral loss of a hexoside moiety (162 Da), obtaining the typical fragmentation of hydroxytyrosol (*m*/*z* 153, 135, and 123). This compound was named as hydroxytyrosol-O-hexoside [20].

Compound **13** was identified as benzyl alcohol hexose pentose (formate adduct) with [M − H]^−^ ion at *m*/*z* 447 and fragments at *m*/*z* 401 (neutral loss of HCl), *m*/*z* 269 (neutral loss of pentoside molecule, 132 Da), and *m*/*z* 161 (neutral loss of benzyl alcohol, 108 Da) corresponding with a hexoside [21].

Compound **18** (*m*/*z* 342) was characterized as magnoflorine using the positive ion mode. This alkaloid presented the base peak at *m*/*z* 297, and other fragment ions at *m*/*z* 282 and 265 [22].

Compound **39**, with [M − H]^−^ ion at *m*/*z* 312, presented fragment ions at *m*/*z* 297, 178, and 135, typical of caffeoyltyramine derivative [23].

Compounds **40** and **41** were identified as oxo-dihydroxy-octadecenoic acid (oxo-DHODE) and trihydroxy-octadecenoic acid (THODE), respectively, based on bibliographic data [24].

### 3.2. Quantification of Phenolic Compounds

Twenty-seven compounds were quantified in the analyzed extracts of *S. enneaphylla*, *S. pulcherrima*, and *S. saetabensis* by HPLC-DAD using analytical standards of the corresponding chemical families.

In the three *Sarcocapnos* species under study, methanolic extracts always presented the highest concentration of phytochemicals, mainly due to the higher solubility of flavonoids in methanol than water. In addition, it implies that methanol is more efficient in cells walls degradation of *Sarcocapnos* species, probably due to their nonpolar character. Quantification data of all the extracts of *Sarcocapnos* are given in Tables 3–5.

Total individual phenolic content (TIPC) was defined as the sum of all the compounds quantified individually by HPLC-DAD. TIPC data are shown in Tables 3–5 as well as in Figure S2 of Supplementary Materials. It can be observed that the TIPC values were in the following order: *S. enneaphylla* > *S. pulcherrima* > *S*. *saetabensis*. This pattern is in agreement with flavonoids concentrations in the three *Sarcocapnos* species.

All extracts had a higher concentration of flavonoids than phenolic acids, except the aqueous extracts of *S. saetabensis*, in which phenolic acids were more abundant. This was due to the presence of compound **19** (caffeic acid cinnamyl ester), which was the dominant phenolic acid. Flavonoids represented a contribution to TIPC of 84–86% (methanol extract) and 53–68% (aqueous extract) in *S. enneaphylla*. For *S*. *pulcherrima*, flavonoids contribution to TIPC was 87% (methanol extract) and 53–61% (aqueous extract). Finally, for *S. saetabensis*, flavonoids contributions to TIPC were 67–77% and 43–46% for the methanol and aqueous extracts, respectively.

The two main flavonoids in all extracts were rutin and isorhamnetin-O-rutinoside. In the methanolic extracts of *S. enneaphylla*, both flavonoids represented 81–93% of the concentration of all flavonoids (80–84% in the aqueous extracts). In *S. pulcherrima*, these two flavonoids represented 61–63% and 65–81% of all flavonoids in the methanol and aqueous extracts, respectively. Finally, in *S. saetabensis*, rutin and isorhamnetin-O-rutinoside accounted for 82–84% and 74–81% of all flavonoids in the methanolic and aqueous extracts, respectively.

The main phenolic acids were 3-feruloylquinic acid isomers, caffeic acid cinnamyl ester, and coumaric acid. These three compounds accounted for the following percentages of TIPC: 74–80% (methanol extract) and 68–70% (aqueous extract) in *S. enneaphylla*, 74–75% (methanol extract) and 59–60% (aqueous extract) in *S. pulcherrima*, and 54–84% (methanol extract) and 55–56% (aqueous extract) in *S. saetabensis*.

### 3.3. Total Phenolic and Flavonoid Contents

**Figure 2:**
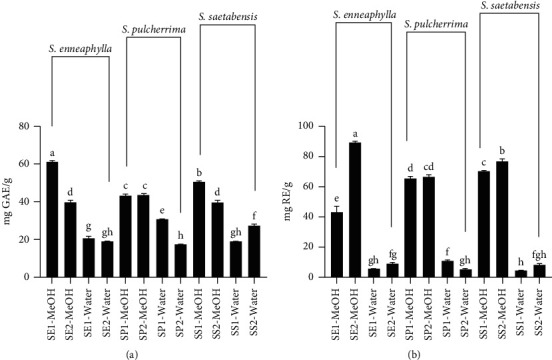
Total phenolic (a) and flavonoid (b) contents of the extracts by spectrophotometric assays. Values are expressed as mean ± S.D. of three parallel measurements. GAE: gallic acid equivalents; RE: rutin equivalents. Different letters indicate significant differences in the extracts (*p* < 0.05).

The total phenolic and flavonoid contents of the *Sarcocapnos* extracts are illustrated in Figure 2 (also in Table S1 of Supplementary Materials). The highest total phenolic content was determined in *S. enneaphylla* (61.2 mg GAE/g), followed by *S. saetabensis* (50.6 mg GAE/g) and *S. pulcherrima* (43.8 mg GAE/g). Regarding the total flavonoid content, the trend was similar to the one for total phenolic content. This finding is consistent with the HPLC-DAD quantification results, which indicated the highest levels of phenolics and flavonoids (particularly rutin) in *S. enneaphylla* methanol extracts. As can be seen in Figure S3 of Supplementary Materials, the levels of total bioactive compounds exhibited significant differences for each *Sarcocapnos* species in terms of location. This fact was also mentioned by several authors who reported that the amounts of bioactive compounds could vary due to climatic of geographical conditions [25–27]. Besides, methanol has proved to be the best solvent for the extraction of phenolics and flavonoids from *Sarcocapnos* species. In earlier studies [28, 29], the effects of solvents on total bioactive compounds were investigated, and methanol was also found to be the most effective for most of them, in agreement with our findings. Based on our information, there are no previous studies concerning the total amount of bioactive compounds in the genus *Sarcocapnos*. In this sense, these results will be a scientific starting point on this genus.

### 3.4. Antioxidant Ability

The antioxidant abilities of the *Sarcocapnos* species are shown in Table 6 and are expressed in terms of standard equivalents (trolox (TE) and EDTA (EDTAE)). We used antioxidant assays involving different mechanisms (metal chelating, radical quenching, and reduction ability). Similar to the levels of total bioactive compounds, antioxidant activities changed in terms of location in each species. Except for metal chelating ability, the tested methanol extracts exhibited stronger antioxidant abilities when compared with water extracts. The best radical scavenging ability in DPPH and ABTS assays was obtained for the methanol extract of *S. enneaphylla* (89 mg TE/g and 136 mg TE/g), followed by *S. saetabensis* (75.3 mg TE/g and 122 mg TE/g) and *S. pulcherrima* (66.8 mg TE/g and 105.3 mg TE/g). CUPRAC and FRAP assays include the transformation of Cu^2+^ to Cu^+^ and Fe^3+^ to Fe^2+^, respectively. These transformations could be important to prevent Fenton or Haber–Weiss reactions and thus could stop the production of hydroxyl radicals. From this point of view, the tested *Sarcocapnos* extracts exhibited significant reduction ability. The highest abilities were recorded in *S. enneaphylla* and *S. saetabansis* methanol extracts, and they were statistically similar (*p* > 0.05). The results for phosphomolybdenum assay, which is based on the transformation of Mo (VI) to Mo (V), were similar to CUPRAC and FRAP, and again the highest ability was noted in *S. enneaphylla* with the value of 1.97 mmol TE/g. The results for radical scavenging and reducing power assays were in good agreement with the levels of total bioactive compounds. Our observations coincide with the previous studies, which found a good analogy between total bioactive compounds and antioxidant properties [30, 31]. Concerning metal chelating ability, the results are inconsistent with the other assays. Metal chelating abilities of the water extracts were found to outperform methanol extracts. This rather contradictory finding may be due to the presence of nonphenolic chelators in the water extracts [32, 33].

### 3.5. Enzyme Inhibition Properties

Several enzymes are targets to manage several diseases, and thus, the symptoms could be decreased by inhibiting these enzymes. Due to the side effects of synthetic compounds, researchers tend to focus on natural sources for the development of novel inhibitors with lower side effects [34–37]. Hence, we investigated the enzyme inhibitory effects of *Sarcocapnos* species against cholinesterases (AChE and BChE), tyrosinase, amylase, and glucosidase. The results are shown in Table 7. Except for glucosidase, all enzymes were inhibited by the tested extracts. Regarding AChE inhibition abilities, the values ranged from 2.7 mg GALAE/g to 5.2 mg GALAE/g, while the values for BChE ranged from 10 mg GALAE/g to 22.2 mg GALAE/g. These inhibition effects may be linked to the chemical profiles of the extracts. For example, the extracts contained some flavonoids such as rutin, isorhamnetin, or kaempferol, which have been previously reported as cholinesterase inhibitors [38–40]. Tyrosinase is a main enzyme in the melanogenesis pathway, and its inhibition is an important therapeutic way to control hyperpigmentation problems. As depicted in Table 7, the methanol extracts exhibited stronger tyrosinase inhibitor effects than water extracts. In the same way, we observed the highest inhibitor effects for methanol extracts against amylase and glucosidase. The tested water extracts were not active on glucosidase. These observations are consistent with the levels of total bioactive compounds. A similar linear correlation between total bioactive components and these inhibitions was reported by other authors [41–44]. In addition, some flavonoids (rutin, isorhamnetin, etc.) have been reported as significant inhibitors against these enzymes [45–48]. Taken together, the tested *Sarcocapnos* species can be appraised as sources of natural enzyme inhibitors to combat global health problems.

### 3.6. Multivariate Analysis

An unsupervised exploratory multivariate analysis PCA was performed on biological activities datasets of *Sarcocapnos* species extracts derived from two solvents with different geographical origins. The main focus of doing PCA was to comprehensively screen the differentially expressed biological activities between *Sarcocapnos* species samples. In fact, two independent factors were involved as follows: geographical origins and solvent used for extraction. Thus, through PCA, we sought to determine which factor was responsible to distinguish between the biological activities of the samples, previously uncovered by the univariate analysis.

**Figure 3:**
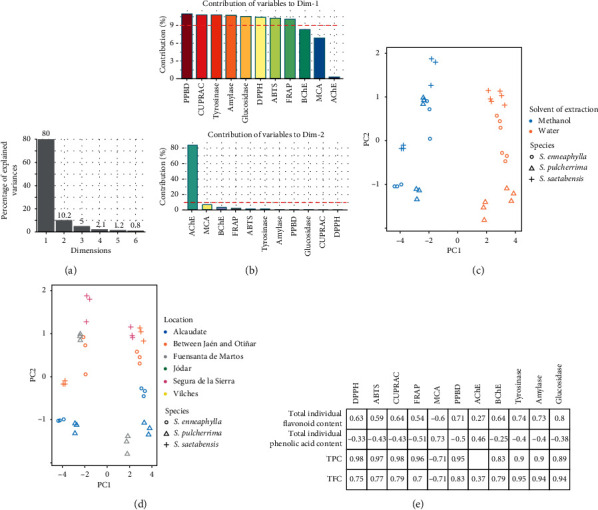
Principal component analysis of *Sarcocapnos* species based on biological activities profiling. (a) Percentage of variance explained by each principal component. (b) Relation between biological activities and the principal component. (c, d) Score plot representing samples in the space PC1 vs PC2 in terms of extractive solvent and geographical origins, respectively. (e) Correlation between phenolic/flavonoid contents and biological activities.

Results are displayed in Figure 3. As seen in Figure 3(a), PC1 and PC2 together expressed a cumulative variance of 90.2% (PC1 = 80%, PC2 = 10.2%). The contribution of biological activities on both retained principal component is shown in Figure 3(b); as can be seen, eight biological activities represented the most associated variables with PC1 while only AChE was most closely linked with PC2. By observing the score plots reported in Figures 3(c) and 3(d), it could be noticed that the extracts can be classified in terms of different factors. Concerning Figure 3(c), extracts were separated into two groups along PC1, based on the solvent used for extraction. Therefore, the biological activities of studied species were affected by the varied polarity of both solvents used. Several reports highlighted the influence of the type of solvent on the biological activities of a large number of species [31, 49, 50]. This disparity reflects the availability of different bioactive compounds, involved in these biological activities, in each extraction solvent. As a result, selecting the appropriate solvent leads to a high recovery yield of molecules of biological interest.

Afterwards, by analyzing the score plots under another angle, the difference between the extracts of *Sarcocapnos* species can be also attributed to a geographical factor (Figure 3(d)). Nonetheless, this geographical factor took effect within methanolic extracts of species. Two subclusters were observed; indeed, samples obtained from specimens of *Sarcocapnos* species from Segura de la Sierra, Vilches, and Fuensanta de Martos, respectively, were distinguished from those harvested in Alcaudete, Jódar, and between Jaén and Otíñar. On the other hand, as regards to the extracts derived from water, a difference was observed only between the samples of *S. enneaphylla.* In more detail, specimen from Vilches was detached from that of Alcaudete along PC1.

**Figure 4:**
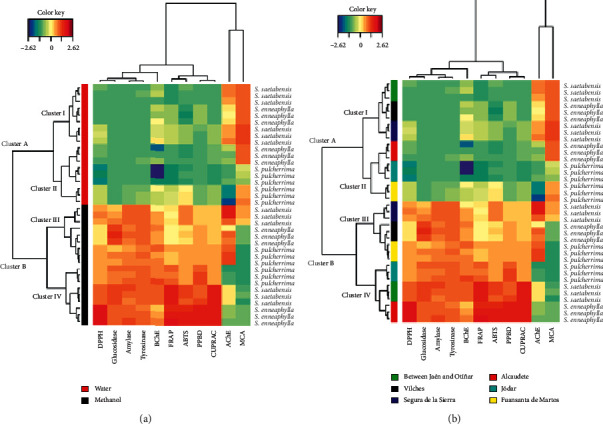
Heatmap and HCA analyses on biological activities of *Sarcocapnos* species built up from PCA result. (a) Clustering in relation to extractive solvent. (b) Clustering in relation to geographical origin. Red color: strong activity. Blue color: low activity.

Heatmap and HCA were produced on the result of PCA taking into account only the first two principal components of PCA, for visualizing differential bioactivities in the different samples (Figure 4). Two major clusters and their respective two subclusters were obtained (Figure 4(a)). As can be seen, methanol extracts of all studied *Sarcocapnos* species were found to be more active against the vast majority of the evaluated biological activities, due to the higher solubility of the bioactive compounds in methanol.

The highest values of total phenolic and total flavonoid contents (spectrophotometric analyses), as well as total individual contents (HPLC-MS), were obtained with extracts derived from methanol for all species. Hence, TPC, TFC, and total individual flavonoids content correlated very well with all biological activities except AChE and MCA (Figure 3(e)). In particular, the total antioxidant activity from DPPH (*R* = 0.75; 0.98; and 0.63), ABTS (*R* = 0.77; 0.97; and 0.59), FRAP (*R* = 0.7; 0.96; 0.54), CUPRAC (*R* = 0.79; 0.98; 0.64), and PPBD (*R* = 083; 0.95; 0.71) assays was positively linked with TFC, TPC, and total individual flavonoid content, respectively, while MCA assay was positively bound to total individual phenolic acid content (*R* = 0.93) at *p* < 0.05. Additionally, the inhibition of tyrosinase, amylase, glucosidase, and BChE was highest with the increasing content of TFC (*R* = 0.95; 0.94; 094; and 0.79), TPC (*R* = 0.9; 0.9; 0.89; 0.93) and total individual flavonoids content (*R* = 0.74; 0.73; 0.8; 0.64) respectively. According to the literature, phenolic compounds (including the flavonoids), ubiquitous in plants, are of great interest due to their considerable beneficial for human health, since they prove to be able to cut down the incidence of several chronic ailments such as diabetes and Alzheimer and skin diseases through their antioxidant properties. Instead, their antioxidant activities are strongly linked with their structure, especially the position and number of the hydroxyl groups, the conjugated double bonds, and the nature of substitutions on the aromatic rings.

Afterwards, by analyzing the score plots under another angle, the difference between the extracts of *Sarcocapnos* species can be also attributed to a geographical factor (Figure 3(d)). Nonetheless, this geographical factor took effect within methanolic extracts of species. Two subclusters were observed; indeed, samples obtained from specimens of *Sarcocapnos* species from Segura de la Sierra, Vilches, and Fuensanta de Martos, respectively, were distinguished from those harvested in Alcaudete, Jódar, and between Jaén and Otíñar. On the other hand, as regards the extracts derived from water, a difference was observed only between the samples of *S. enneaphylla.* In more detail, specimen from Vilches was detached from that of Alcaudete along PC1.

Regarding Figure 4(b), the difference across subclusters III and IV as well as between *S. enneaphylla* specimens was found tangibly with AChE inhibition assay. As it happens, the highest activity was obtained within cluster III and *S. enneaphylla* specimen from Vilches (belonging the cluster I), respectively.

With regard to our results, the biological activities of studied species varied among the geographical origins. This observation is closely linked to the presence/absence of some key bioactive compounds under the influence of the variation of climatic conditions as well as the soil nutrient composition. This is in agreement with the report of Rocchetti [51], who underlined the impact of different geographical origins on phenolic composition of chardonnay wines.

## 4. Conclusion

In this work, we have presented the first report on the phenolic composition of three *Sarcocapnos* species as well as the relation with the bioactive properties. For the three species, methanol was the most suitable solvent, and the highest phenolic content as well as the highest bioactivities were found in extracts from *S. enneaphylla*, although the other species were also active in all the assays. Flavonoids were the most abundant compounds, followed by phenolic acids. Among flavonoids, rutin, kaempferol, and isorhamnetin glycosides were the main compounds. These compounds have been previously reported to present significant bioactivity and, thus, may be the main contributors to the observed effects. Multivariate analysis confirmed the relationship between phenolics concentration and bioactivity. It also indicated a clear difference within the same species depending on the geographical origin. The results here presented may open new ways to valorize *Sarcocapnos* species as sources of natural compounds for the food (nutraceuticals or food supplements) or pharmaceutical industry and represent a starting point for additional research, being the final goal the industrial applications of *Sarcocapnos* species.

## Figures and Tables

**Table 1:** *Sarcocapnos* species collected and their location.

*Sarcocapnos* species	Sample	Location	Geographical coordinates/altitude (m a.s.l.)	Herbarium sheet
*S. enneaphylla*	SE1	Alcaudete (Jaén, Spain)	37°36′5.39″N 4°2′56.57″W/891	GDA 65532
	SE2	Vilches (Jaén, Spain)	38°12′ 52.4″N 3°28′53.8″W/690	GDA 65525

*S. pulcherrima*	SP1	Fuensanta de Martos (Jaén, Spain)	37°38′59.6″N 3°53′57.9″W/796	GDA 65529, GDA 65530
	SP2	Jódar (Jaén, Spain)	37°48′47.9″N 3°20′59.9″W/1020	GDA 65531

*S. saetabensis*	SS1	Between Jaén and Otíñar (Jaén, Spain)	37°41′28.3″N 3°45′46.2″W/606	GDA 65536
	SS2	Segura de la Sierra (Jaén, Spain)	38°17′54.9″N 2°39′00.6″W/1202	GDA 65541, GDA 65542

m.a.s.l.: metres above sea level; GDA: Herbarium of the University of Granada.

**Table 2:** Characterization of the compounds found in the analyzed extracts of *Sarcocapnos* species by HPLC-DAD/ESI-MS^n^, in methanolic (M) and aqueous (W) media.

No.	*t*_*R*_ (min)	[M − H]^−^*m*/*z*	*m*/*z* (% base peak)	Assigned identification	*S. enneaphylla*				*S. pulcherrima*				*S. saetabensis*			
					SE1		SE2		SP1		SP2		SS1		SS2	
					M	W	M	W	M	W	M	W	M	W	M	W
**1**	1.8	377	MS^2^ [377]: 341 (100), 179 (9)	Disaccharide (HCl adduct)	✓		✓				✓		✓			
			MS^3^ [377 ⟶ 341]: 179 (100), 161 (17), 143 (15)													
			MS^4^ [377 ⟶ 341 ⟶ 179]: 161 (100), 143 (57), 131 (65)													

**2**	2.0	191	MS^2^ [191]: 173 (100), 155 (5), 111 (95)	Isocitric acid		✓		✓	✓	✓		✓		✓	✓	✓
			MS^3^ [191 ⟶ 173]: 155 (9), 111 (100)													

**3**	2.2	367	MS^2^ [367]: 193 (100), 134 (10)	3-Feruloylquinic acid isomer			✓		✓		✓		✓		✓	
			MS^3^ [367 ⟶ 193]: 149 (45), 134 (100)													

**4**	2.5	191	MS^2^ [191]: 173 (51), 111 (100)	Citric acid		✓		✓		✓	✓	✓		✓	✓	✓

**5**	2.9	564	MS^2^ [564]: 293 (7), 271 (13), 270 (100), 162 (24)	Unknown	✓	✓				✓		✓	✓	✓		✓
			MS^3^ [564 ⟶ 270]: 163 (9), 162 (100), 147 (2)													

**6**	3.6	315	MS^2^ [315]: 153 (100), 135 (89), 123 (26)	Hydroxytyrosol-O-hexoside						✓	✓	✓	✓	✓	✓	✓
			MS^3^ [315 ⟶ 153]: 123 (100)													

**7**	3.8	477	MS^2^ [477]: 431 (100), 293 (78)	Unknown	✓	✓	✓	✓				✓	✓	✓	✓	✓
			MS^3^ [477 ⟶ 431]: 293 (100), 191 (13), 125 (9)													
			MS^4^ [477 ⟶ 431 ⟶ 293]: 126 (100), 125 (8)													

**8**	5.2	353	MS^2^ [353]: 191 (100), 179 (60), 135 (12)	Neochlorogenic acid^*∗*^		✓		✓		✓		✓		✓	✓	✓

**9**	7.4	337	MS^2^ [337]: 191 (11), 163 (100)	3-p-Coumaroylquinic acid isomer					✓	✓	✓	✓				
			MS^3^ [337 ⟶ 163]: 119 (100)													

**10**	7.8	337	MS^2^ [337]: 191 (24), 163 (100)	3-p-Coumaroylquinic acid isomer					✓	✓	✓	✓				
			MS^3^ [337 ⟶ 163]: 119 (100)													

**11**	9.0	367	MS^2^ [367]: 193 (100), 134 (10)	3-Feruloylquinic acid isomer	✓	✓	✓	✓	✓	✓	✓	✓	✓	✓	✓	✓
			MS^3^ [367 ⟶ 193]: 149 (20), 134 (100)													

**12**	9.3	771	MS^2^ [771]: 609 (100), 301 (8)	Quercetin-O-hexoside-O-rutinoside			✓	✓						✓		✓
			MS^3^ [771 ⟶ 609]: 301 (100)													
			MS^4^ [771 ⟶ 609 ⟶ 301]: 271 (57), 179 (100), 151 (39)													

**13**	9.5	447	MS^2^ [447]: 401 (100), 269 (11)	Benzyl alcohol hexose pentose (formate adduct)					✓	✓	✓	✓				
			MS^3^ [447 ⟶ 401]: 269 (100), 161 (19)													
			MS^4^ [447 ⟶ 401 ⟶ 269]: 161 (100), 143 (16), 113 (12)													

**14**	10.1	369	MS^2^ [369]: 193 (100), 178 (7), 175 (62), 113 (39)	Ferulic acid glucuronide				✓						✓		✓
			MS^3^ [369 ⟶ 193]: 134 (100)													

**15**	10.8	506	MS^2^ [506]: 460 (100), 413 (29), 293 (37)	Unknown	✓	✓	✓	✓	✓	✓	✓	✓	✓	✓	✓	✓
			MS^3^ [506 ⟶ 460]: 413 (78), 293 (100)													
			MS^4^ [506 ⟶ 460 ⟶ 293]: 191 (29), 149 (100), 131 (68)													

**16**	12.2	367	MS^2^ [367]: 193 (11), 173 (100)	4-Feruloylquinic acid isomer				✓		✓		✓		✓		✓
			MS^3^ [367 ⟶ 173]: 111 (100)													

**17**	12.6	785	MS^2^ [785]: 623 (100), 315 (2)	Isorhamnetin-O-rutinoside-O-hexoside				✓						✓		✓
			MS^3^ [785 ⟶ 623]: 315 (100), 300 (24)													
			MS^4^ [785 ⟶ 623 ⟶ 315]: 300 (100)													

**18**	12.8	342 (+)	MS^2^ [342]: 297 (100), 282 (16), 279 (24), 265 (83)	Magnoflorine	✓	✓			✓	✓	✓		✓	✓	✓	✓
			MS^3^ [342 ⟶ 297]: 282 (14), 265 (100), 237 (11)													
			MS^4^ [342 ⟶ 297 ⟶ 265]: 250 (28), 237 (100), 205 (24)													

**19**	13.3	295	MS^2^ [295]: 179 (100), 135 (30), 133 (59)	Caffeic acid cinnamyl ester	✓	✓	✓	✓		✓		✓		✓	✓	✓
			MS^3^ [295 ⟶ 179]: 135 (100)													

**20**	13.7	367	MS^2^ [367]: 193 (11), 173 (100)	4-Feruloylquinic acid isomer	✓	✓	✓		✓	✓		✓	✓	✓	✓	✓

**21**	13.7	771	MS^2^ [771]: 301 (100)	Quercetin-deoxyhexoside-hexoside-hexoside				✓			✓					
			MS^3^ [771 ⟶ 301]: 179 (100)													

**22**	14.3	367	MS^2^ [367]: 191 (100), 173 (5)	5-Feruloylquinic acid isomer		✓		✓		✓		✓		✓		✓
			MS^3^ [367 ⟶ 191]: 134 (100)													

**23**	15.2	453	MS^2^ [453]: 407 (100), 163 (9)	Coumaric acid-O-hexoside derivative (formate adduct)			✓								✓	✓
			MS^3^ [453 ⟶ 407]: 325 (18), 163 (100)													
			MS^4^ [453 ⟶ 407 ⟶ 163]: 119 (100)													

**24**	16.2	367	MS^2^ [367]: 191 (100), 173 (2)	5-Feruloylquinic acid isomer				✓						✓		✓
			MS^3^ [367 ⟶ 191]: 127 (100)													

**25**	16.4	785	MS^2^ [785]:623 (100)	Isorhamnetin-O-rutinoside-O-hexoside			✓		✓	✓	✓	✓				
			MS^3^ [785 ⟶ 623]: 315 (100), 300 (10), 255 (16)													

**26**	18.1	163	MS^2^ [163]: 119 (100)	Coumaric acid^*∗*^		✓	✓	✓	✓	✓	✓	✓		✓	✓	✓

**27**	19.4	609	MS^2^ [609]: 301 (100)	Rutin^*∗*^	✓	✓	✓	✓	✓	✓	✓	✓	✓	✓	✓	✓
			MS^3^ [609 ⟶ 301]: 179 (100), 151 (43)													
			MS^4^ [609 ⟶ 301 ⟶ 179]: 151 (100)													

**28**	19.8	193	MS^2^ [193]: 149 (80), 134 (100)	Ferulic acid^*∗*^	✓	✓	✓	✓	✓	✓	✓	✓	✓	✓	✓	✓

**29**	20.5	559	MS^2^ [559]: 443 (100), 327 (61)	Coumaric acid derivative								✓				
			MS^3^ [559 ⟶ 443]: 327 (100), 283 (2)													
			MS^4^ [559 ⟶ 443 ⟶ 327]: 283 (34), 239 (67), 163 (100), 119 (35)													
**30**	20.6	463	MS^2^ [463]: 301 (100)	Quercetin-O-hexoside			✓	✓								
			MS^3^ [463 ⟶ 301]: 179 (100), 151 (52)													
			MS^4^ [463 ⟶ 301 ⟶ 179]: 151 (100)													

**31**	21.4	593	MS^2^ [593]: 285 (100)	Kaempferol-O-rutinoside	✓	✓	✓	✓	✓	✓			✓	✓	✓	✓
			MS^3^ [593 ⟶ 285]: 255 (100)													

**32**	21.8	683	MS^2^ [683]: 521 (42), 367 (100), 315 (36)	Sinapic acid derivative	✓	✓	✓	✓							✓	✓
			MS^3^ [683 ⟶ 367]: 223 (46), 205 (100)													

**33**	22.6	593	MS^2^ [593]: 285 (100)	Kaempferol-O-rutinoside	✓	✓	✓	✓	✓	✓	✓	✓	✓	✓	✓	✓
			MS^3^ [593 ⟶ 285]: 257 (100)													

**34**	22.8	623	MS^2^ [623]: 315 (100)	Isorhamnetin-O-rutinoside	✓	✓	✓	✓	✓	✓	✓	✓	✓	✓	✓	✓
			MS^3^ [623 ⟶ 315]: 300 (100)													
			MS^4^ [623 ⟶ 315 ⟶ 300]: 271 (100), 255 (77)													

**35**	23.3	623	MS^2^ [623]: 315 (100)	Isorhamnetin-O-rutinoside	✓	✓	✓	✓	✓	✓	✓	✓	✓	✓	✓	✓
			MS^3^ [623 ⟶ 315]: 300 (100)													
			MS^4^ [623 ⟶ 315 ⟶ 300]: 271 (41), 255 (100)													

**36**	24.1	477	MS^2^ [477]: 315 (100)	Isorhamnetin-O-hexoside					✓	✓	✓					
			MS^3^ [477 ⟶ 315]: 300 (100), 285 (82)													

**37**	26.3	448	MS^2^ [448]: 404 (52), 360 (100)	Unknown			✓		✓				✓		✓	
			MS^3^ [448 ⟶ 360]: 342 (90), 314 (100)													
			MS^4^ [448 ⟶ 360 ⟶ 314]: 287 (100)													

**38**	26.8	519	MS^2^ [519]: 315 (100)	Isorhamnetin-O-acetylhexoside					✓		✓					
			MS^3^ [519 ⟶ 315]: 300 (100)													
			MS^4^ [519 ⟶ 315 ⟶ 300]: 271 (100), 255 (51)													

**39**	31.5	312	MS^2^ [312]: 297 (70), 178 (100), 135 (60)	Caffeoyltyramine derivative	✓		✓	✓	✓	✓	✓	✓	✓		✓	✓

**40**	39.1	327	MS^2^ [327]: 229 (100), 211 (35), 171 (42)	Oxo-dihydroxy-octadecenoic acid	✓	✓	✓	✓	✓	✓					✓	✓
			MS^3^ [327 ⟶ 229]: 211 (100), 209 (78), 183 (23)													

**41**	40.6	329	MS^2^ [329]: 311 (37), 293 (33), 229 (100), 211 (90), 171 (15)	Trihydroxy-octadecenoic acid	✓	✓	✓		✓	✓			✓		✓	✓
			MS^3^ [329 ⟶ 229]: 211 (100), 209 (65), 183 (36), 125 (17)													

^*∗*^Identified with analytical standards.

**Table 3:** Quantification of compounds in extracts of *S. enneaphylla* (SE) from two different locations, extracted in methanol and water.

Compound		*S. enneaphylla*			
		MeOH		H_2_O	
		SE1	SE2	SE1	SE2
*Flavonoids*					
**12**	Quercetin-O-hexoside-O-rutinoside	—	0.39 ± 0.02	—	0.93 ± 0.05
**17**	Isorhamnetin-O-rutinoside-O-hexoside	—	—	—	0.28 ± 0.01
**21**	Quercetin-deoxyhexoside-hexoside-hexoside	—	—	—	1.11 ± 0.06
**25**	Isorhamnetin-O-rutinoside-O-hexoside	—	0.28 ± 0.01	—	—
**27**	Rutin	14.7 ± 0.7	35 ± 2	4.4 ± 0.2	10.5 ± 0.4
**30**	Quercetin-O-hexoside	—	0.36 ± 0.02	—	0.15 ± 0.01
**31**	Kaempferol-O-rutinoside	0.87 ± 0.04	0.30 ± 0.02	0.27 ± 0.01	0.15 ± 0.01
**33** **+** **34**	Kaempferol-O-rutinoside + Isorhamnetin-O-rutinoside	5.4 ± 0.2	2.5 ± 0.1	1.18 ± 0.05	0.66 ± 0.03
**35**	Isorhamnetin-O-rutinoside	12.6 ± 0.5	10.5 ± 0.5	3.2 ± 0.2	2.9 ± 0.2
Total		**33.6** **±** **0.9**	**49** **±** **2**	**9.1** **±** **0.3**	**16.7** **±** **0.5**
*Phenolic acids*					
**3**	3-Feruloylquinic acid isomer	—	0.70 ± 0.04	—	—
**8**	Neochlorogenic acid	—	—	0.47 ± 0.02	0.61 ± 0.03
**11**	3-Feruloylquinic acid isomer	3.0 ± 0.1	4.9 ± 0.2	0.92 ± 0.05	1.53 ± 0.08
**14**	Ferulic acid glucuronide	—	—	—	0.080 ± 0.003
**16**	4-Feruloylquinic acid isomer	—	—	—	0.14 ± 0.01
**19**	Caffeic acid cinnamyl ester	1.32 ± 0.04	1.78 ± 0.08	4.25 ± 0.2	3.7 ± 0.2
**20**	4-Feruloylquinic acid isomer	0.20 ± 0.01	0.74 ± 0.03	0.56 ± 0.03	—
**22**	5-Feruloylquinic acid isomer	—	—	0.42 ± 0.02	0.46 ± 0.03
**24**	5-Feruloylquinic acid isomer	—	—	—	0.10 ± 0.01
**26**	Coumaric acid	—	0.22 ± 0.01	0.37 ± 0.02	0.27 ± 0.01
**28**	Ferulic acid	0.74 ± 0.04	0.83 ± 0.04	0.99 ± 0.05	0.94 ± 0.05
**32**	Sinapic acid derivative	0.160 ± 0.007	0.120 ± 0.06	0.13 ± 0.01	0.100 ± 0.008
Total		**5.4** **±** **0.1**	**9.3** **±** **0.2**	**8.1** **±** **0.2**	**7.9** **±** **0.2**
TIPC		**39.0** **±** **0.9**	**58** **±** **2**	**17.2** **±** **0.4**	**24.6** **±** **0.5**

Values (mg/g DE) are mean ± SD of three parallel measurements. TIPC: total individual phenolic content.

**Table 4:** Quantification of compounds in extracts of *S. pulcherrima* (SP) from two different locations, extracted in methanol and water.

Compound		*S. pulcherrima*			
		MeOH		H_2_O	
		SP1	SP2	SP1	SP2
*Flavonoids*					
**21**	Quercetin-deoxyhexoside-hexoside-hexoside	—	0.77 ± 0.03	—	—
**25**	Isorhamnetin-O-rutinoside-O-hexoside	0.39 ± 0.02	0.97 ± 0.05	0.35 ± 0.02	0.85 ± 0.05
**27**	Rutin	6.9 ± 0.3	4.3 ± 0.2	3.5 ± 0.2	2.0 ± 0.1
**31**	Kaempferol-O-rutinoside	0.62 ± 0.03	—	0.31 ± 0.01	—
**33** **+** **34**	Kaempferol-O-rutinoside + Isorhamnetin-O-rutinoside	9.4 ± 0.5	5.1 ± 0.3	1.17 ± 0.07	1.56 ± 0.07
**35**	Isorhamnetin-O-rutinoside	11.5 ± 0.6	6.8 ± 0.4	5.1 ± 0.2	2.5 ± 0.1
**36**	Isorhamnetin-O-hexoside	0.30 ± 0.02	0.15 ± 0.01	0.16 ± 0.01	—
**38**	Isorhamnetin-O-acetylhexoside	0.23 ± 0.01	0.17 ± 0.02	—	—
Total		**29.3** **±** **0.8**	**18.3** **±** **0.5**	**10.6** **±** **0.3**	**6.9** **±** **0.2**
*Phenolic acids*					
**3**	3-Feruloylquinic acid isomer	0.18 ± 0.01	0.12 ± 0.01	—	—
**8**	Neochlorogenic acid	—	—	0.37 ± 0.02	0.37 ± 0.02
**9**	3-p-Coumaroylquinic acid isomer	0.18 ± 0.01	0.15 ± 0.01	0.13 ± 0.01	0.13 ± 0.01
**10**	3-p-Coumaroylquinic acid isomer	0.27 ± 0.02	0.22 ± 0.01	0.19 ± 0.01	0.19 ± 0.02
**11**	3-Feruloylquinic acid isomer	2.0 ± 0.2	1.4 ± 0.05	1.07 ± 0.06	0.84 ± 0.04
**16**	4-Feruloylquinic acid isomer	—	—	0.23 ± 0.02	0.20 ± 0.01
**19**	Caffeic acid cinnamyl ester	—	—	0.83 ± 0.04	0.94 ± 0.04
**20**	4-Feruloylquinic acid isomer	0.22 ± 0.01	—	0.82 ± 0.03	0.76 ± 0.03
**22**	5-Feruloylquinic acid isomer	—	—	0.33 ± 0.02	0.21 ± 0.01
**26**	Coumaric acid	1.27 ± 0.05	0.66 ± 0.04	1.91 ± 0.08	1.75 ± 0.09
**28**	Ferulic acid	0.28 ± 0.01	0.18 ± 0.01	0.61 ± 0.03	0.50 ± 0.03
Total		**4.4** **±** **0.2**	**2.73** **±** **0.07**	**6.5** **±** **0.1**	**5.9** **±** **0.1**
*Other compounds*					
**6**	Hydroxytyrosol-O-hexoside	—	0.090 ± 0.004	0.100 ± 0.003	0.080 ± 0.003
TIPC		**33.7** **±** **0.8**	**21.1** **±** **0.5**	**17.2** **±** **0.3**	**12.9** **±** **0.2**

Values (mg/g DE) are mean ± SD of three parallel measurements. TIPC: total individual phenolic content.

**Table 5:** Quantification of compounds in extracts of *S. saetabensis* (SS) from two different locations, extracted in methanol and water.

Compound		*S. saetabensis*			
		MeOH		H_2_O	
		SS1	SS2	SS1	SS2
*Flavonoids*					
**12**	Quercetin-O-hexoside-O-rutinoside	—	—	0.46 ± 0.02	0.35 ± 0.02
**17**	Isorhamnetin-O-rutinoside-O-hexoside	—	—	0.21 ± 0.01	0.31 ± 0.01
**27**	Rutin	11.7 ± 0.5	8.9 ± 0.4	3.59 ± 0.02	4.5 ± 0.3
**31**	Kaempferol-O-rutinoside	0.60 ± 0.03	0.32 ± 0.02	0.20 ± 0.01	0.20 ± 0.01
**33** **+** **34**	Kaempferol-O-rutinoside + Isorhamnetin-O-rutinoside	2.8 ± 0.1	2.84 ± 0.01	0.75 ± 0.04	1.16 ± 0.05
**35**	Isorhamnetin-O-rutinoside	3.5 ± 0.2	8.4 ± 0.4	1.03 ± 0.04	3.9 ± 0.2
Total		**18.6** **±** **0.5**	**20.5** **±** **0.6**	**6.24** **±** **0.06**	**10.4** **±** **0.4**
*Phenolic acids*					
**3**	3-Feruloylquinic acid isomer	—	1.01 ± 0.06	—	—
**8**	Neochlorogenic acid	—	2.5 ± 0.2	0.78 ± 0.04	2.1 ± 0.1
**11**	3-Feruloylquinic acid isomer	4.1 ± 0.2	3.5 ± 0.2	1.51 ± 0.07	2.1 ± 0.1
**14**	Ferulic acid glucuronide	—	—	0.100 ± 0.006	0.10 ± 0.01
**16**	4-Feruloylquinic acid isomer	—	—	0.20 ± 0.01	0.27 ± 0.02
**19**	Caffeic acid cinnamyl ester	—	1.38 ± 0.05	2.7 ± 0.2	4.0 ± 0.2
**20**	4-Feruloylquinic acid isomer	0.19 ± 0.01	0.24 ± 0.01	1.00 ± 0.04	1.06 ± 0.03
**22**	5-Feruloylquinic acid isomer	—	—	0.50 ± 0.03	0.56 ± 0.03
**24**	5-Feruloylquinic acid isomer	—	—	0.090 ± 0.004	0.090 ± 0.004
**26**	Coumaric acid	—	0.32 ± 0.01	0.26 ± 0.01	0.43 ± 0.02
**28**	Ferulic acid	0.58 ± 0.03	0.56 ± 0.03	0.84 ± 0.05	0.98 ± 0.04
**32**	Sinapic acid derivative	—	0.15 ± 0.01	—	0.12 ± 0.01
Total		**4.9** **±** **0.2**	**9.7** **±** **0.3**	**8.0** **±** **0.2**	**11.8** **±** **0.3**
*Other compounds*					
**6**	Hydroxytyrosol-O-hexoside	0.59 ± 0.03	0.25 ± 0.01	0.28 ± 0.01	0.24 ± 0.01
TIPC		**24.1** **±** **0.5**	**30.5** **±** **0.7**	**14.5** **±** **0.2**	**22.4** **±** **0.5**

Values (mg/g DE) are mean ± SD of three parallel measurements. TIPC: total individual phenolic content.

**Table 6:** Antioxidant activities of the extracts of *S. enneaphylla* (SE), *S. pulcherrima* (SP), and *S. saetabensis* (SS), each one from two different locations, extracted in methanol and water.

Samples			DPPH (mg TE/g)	ABTS (mg TE/g)	CUPRAC (mg TE/g)	FRAP (mg TE/g)	Phosphomolybdenum (mmol TE/g)	Chelating activity (mg EDTAE/g)
*S. enneaphylla*	MeOH	SE1	89 ± 1^a^	136 ± 3^a^	289 ± 9^a^	165 ± 9^a^	1.97 ± 0.02^a^	28.3 ± 0.5^d^
		SE2	60 ± 1^d^	95 ± 4^d^	182 ± 3^d^	101 ± 1^d^	1.26 ± 0.06^c^	28.2 ± 0.7^d^
	H_2_O	SE1	38.2 ± 0.4^f^	50.8 ± 0.7^fg^	76 ± 2^f^	61.1 ± 0.9^g^	0.11 ± 0.04^gh^	64.3 ± 0.3^a^
		SE2	36 ± 1^f^	46 ± 2^g^	66.9 ± 0.8^g^	75.2 ± 0.4^f^	0.35 ± 0.01^e^	62.3 ± 0.9^a^

*S. pulcherrima*	MeOH	SP1	66.8 ± 0.6^c^	105.3 ± 0.7^c^	199 ± 6^b^	118 ± 4^c^	1.27 ± 0.04^c^	18.8 ± 0.4^f^
		SP2	67 ± 1^c^	107 ± 2^c^	195 ± 1^bc^	139 ± 3^b^	1.63 ± 0.01^b^	20 ± 1^ef^
	H_2_O	SP1	48 ± 1^e^	92 ± 3^d^	98 ± 2^e^	89 ± 3^e^	0.23 ± 0.04^f^	56.9 ± 0.2^b^
		SP2	26 ± 2^g^	51.2 ± 0.6^fg^	51.8 ± 0.2^h^	44.7 ± 0.3^h^	0.03 ± 0.01^h^	36 ± 4^c^

*S. saetabensis*	MeOH	SS1	75.3 ± 0.6^b^	122 ± 3^b^	288.3 ± 0.8^a^	165.7 ± 0.7^a^	1.59 ± 0.04^b^	24 ± 3^de^
		SS2	69 ± 1^c^	112 ± 0.9^c^	187.5 ± 0.6^cd^	105 ± 3^d^	1.11 ± 0.01^d^	55 ± 2^b^
	H_2_O	SS1	37.9 ± 0.6^f^	56 ± 3^f^	59.2 ± 0.5^gh^	53.3 ± 0.2^gh^	0.12 ± 0.03^gh^	62.7 ± 0.2^a^
		SS2	49.2 ± 0.8^e^	66 ± 1^e^	95 ± 1^e^	80 ± 0.6^ef^	0.16 ± 0.04^fg^	65.9 ± 0.6^a^

Values expressed are means ± S.D. of three parallel measurements; TE: Trolox equivalent; EDTAE: EDTA equivalent. Different letters indicate significant differences in the extracts (*p* < 0.05).

**Table 7:** Enzyme inhibitory properties of the extracts of *S. enneaphylla* (SE), *S. pulcherrima* (SP), and *S. saetabensis* (SS), each one from two different locations, extracted in methanol and water.

Samples			AChE inhibition (mg GALAE/g)	BChE inhibition (mg GALAE/g)	Tyrosinase inhibition (mg KAE/g)	Amylase inhibition (mmol ACAE/g)	Glucosidase inhibition (mmol ACAE/g)
*S. enneaphylla*	MeOH	SE1	3.50 ± 0.03^d^	20.8 ± 0.9^abc^	143 ± 3^a^	0.61 ± 0.03^a^	2.82 ± 0.01^ab^
		SE2	4.5 ± 0.4^c^	20.8 ± 0.3^abc^	140.5 ± 0.2^a^	0.55 ± 0.03^ab^	3.11 ± 0.03^a^
	H2O	SE1	3.6 ± 0.2^d^	13 ± 2^fg^	42 ± 5^c^	0.08 ± 0.01^c^	na
		SE2	4.18 ± 0.07^c^	16 ± 1^def^	43 ± 3^c^	0.08 ± 0.01^c^	na

*S. pulcherrima*	MeOH	SP1	4.97 ± 0.07^ab^	21.7 ± 0.8^ab^	140 ± 3^a^	0.51 ± 0.01^b^	2.1 ± 0.3^c^
		SP2	3.2 ± 0.1^d^	22.0 ± 0.6^ab^	142.46 ± 0.08^a^	0.57 ± 0.04^ab^	2.5 ± 0.2^b^
	H2O	SP1	2.7 ± 0.2^e^	17.3 ± 0.8^cdef^	57 ± 5^b^	0.10 ± 0.01^c^	na
		SP2	3.28 ± 0.04^d^	10 ± 3^g^	39 ± 2^c^	0.09 ± 0.01^c^	na

*S. saetabensis*	MeOH	SS1	4.19 ± 0.01^c^	22.2 ± 0.2^a^	144.2 ± 0.8^a^	0.59 ± 0.06^a^	2.87 ± 0.01^ab^
		SS2	5.2 ± 0.3^a^	20 ± 2^abcd^	142.2 ± 0.8^a^	0.59 ± 0.01^a^	2.1 ± 0.2^c^
	H2O	SS1	4.64 ± 0.07^bc^	15 ± 1^ef^	44 ± 3^c^	0.08 ± 0.01^c^	na
		SS2	4.60 ± 0.07^bc^	18 ± 1^bcde^	46.2 ± 0.7^c^	0.08 ± 0.01^c^	na

Values expressed are mean ± S.D. of three parallel measurements; AChE: acetylcholinesterase; BChE: butyrylcholinesterase; GALAE: galantamine equivalent; KAE: kojic acid equivalent; ACAE: acarbose equivalent; na: not active. Different letters indicate significant differences in the extracts (*p* < 0.05).

## Data Availability

The data used to support the findings of this study are included within the article.

## Supplementary Materials

Supplementary Materials File S1: chemicals and reagents. File S2: HPLC analysis conditions. File S3: assays for total phenolic and flavonoid contents. File S4: determination of antioxidant and enzyme inhibitory effects. Table S1: total phenolic and flavonoid contents of the extracts of *S. enneaphylla* (SE), *S. pulcherrima* (SP), and *S. saetabensis* (SS), each one from two different locations, extracted in methanol and water. Figure S1: HPLC-ESI/MS^n^ base peak chromatograms (BPC) of the aqueous extracts of (A) *S. enneaphylla* (SE1), (B) *S. pulcherrima* (SP1), and (C) *S. saetabensis* (SS2). Figure S2: ranges of flavonoids, phenolic acids, and TIPC values for the three *Sarcocapnos* species. Figure S3: total individual phenolic content (A), total flavonoid content (B), and phenolic acid content (C) of the extracts by HPLC-DAD quantification. Different letters indicate significant differences in the extracts (*p* < 0.05).
