# Supplementary material for: Study on Three Sarcocapnos Species as Potential Sources of Bioactive Compounds: Relation between Phenolic Content and Bioactivity by Multivariate Analysis
Source: J Anal Methods Chem. 2020 Jul 11;2020:8885169. doi: 10.1155/2020/8885169 (PMC7369672; doi:10.1155/2020/8885169)
Supplement: Supplementary Materials — File S1: chemicals and reagents. File S2: HPLC analysis conditions. File S3: assays for total phenolic and flavonoid contents. File S4: determination of antioxidant and enzyme inhibitory effects. Table S1: total phenolic and flavonoid contents of the extracts of S. enneaphylla (SE), S. pulcherrima (SP), and S. saetabensis (SS), each one from two different locations, extracted in methanol and water. Figure S1: HPLC-ESI/MSn base peak chromatograms (BPC) of the aqueous extracts of (A) S. enneaphylla (SE1), (B) S. pulcherrima (SP1), and (C) S. saetabensis (SS2). Figure S2: ranges of flavonoids, phenolic acids, and TIPC values for the three Sarcocapnos species. Figure S3: total individual phenolic content (A), total flavonoid content (B), and phenolic acid content (C) of the extracts by HPLC-DAD quantification. Different letters indicate significant differences in the extracts (p < 0.05). [file 8885169.f1.docx]

**Study on three *Sarcocapnos* species as potential sources of bioactive compounds: relation between phenolic content and bioactivity by multivariate analysis**

María del Pilar Fernández-Poyatos^1^, Gökhan Zengin^2^, Carlos Salazar-Mendías^3^, Antonio Ruiz-Medina^1^, Kouadio Ibrahime Sinan^2^, and Eulogio J. Llorent-Martínez^1^

*^1^Department of Physical and Analytical Chemistry, Faculty of Experimental Sciences, University of Jaén, Campus Las Lagunillas, E-23071 Jaén, Spain*

*^2^Department of Biology, Science Faculty, Selcuk University, Campus, Konya, Turkey*

*^3^Department of Animal Biology, Plant Biology and Ecology, Faculty of Experimental Sciences, University of Jaén, Campus Las Lagunillas, E-23071 Jaén, Spain.*

**Supplementary Materials**

**File S1: Chemicals and Reagents**

Chemicals and reagents (analytical grade) were purchased from Sigma-Aldrich. They were: analytical standards for phenolic compounds (caffeic acid, neochlorogenic acid, coumaric acid, ferulic acid, hidroxityrosol, sinapic acid, quercetin, kaempferol, and rutin). 2,2´-azino-bis(3-ethylbenzothiazoline-6-sulphonic acid (ABTS), 2,2-diphenyl-1-picrylhydrazyl (DPPH), 2,4,6-Tris(2-pyridyl)-s-triazine (TPTZ), 3,4-dihydroxy-L-phenylala-nine (L-DOPA), 4-N-trophenyl-α-D-glucopyranoside (PNPG), 5,5-dithio-bis(2-nitrobenzoic) acid (DTNB), 6-hydroxy-2,5,7,8-tetramethylchroman-2-carboxylic acid (Trolox), acarbose, acetonitrile (LC-MS grade), acetylthiocholine iodide (ATCI), aluminium chloride, ammonium acetate, ammonium molybdate, amylase (EC. 3.2.1.1, from porcine pancreas), butyrylthiocholine chloride (BTCI), cupric chloride, electric eel acetylcholinesterase (AChE) (type-VI-S, EC 3.1.1.7), ethylenediaminetetraacetate (EDTA), ferric chloride, ferrous sulphate hexahydrate, ferrozine, Folin-Ciocalteu reagent, formic acid, gallic acid, galantamine, glucosidase (EC. 3.2.1.20, from Saccharomyces cerevisiae), glutathione, horse serum butyrylcholinesterase (BChE) (EC 3.1.1.8), hydrochloric acid, iodine-potassium iodide, kojic acid, methanol (HPLC grade), LC-MS grade acetonitrile, neocuproine, potassium persulfate, sodium carbonate, sodium phosphate, starch, sulfuric acid, and tyrosinase (EC1.14.18.1, mushroom). Ultrapure water was also used.

**File S2: HPLC Analysis Conditions**

The HPLC system was an Agilent Series 1100, composed of a vacuum degasser, an autosampler, a binary pump, and a G1315B diode array detector (Agilent Technologies, Santa Clara, CA, USA). We used a reversed phase Luna Omega Polar C_18_ analytical column of 150 x 3.0 mm and 5 µm particle size (Phenomenex, Torrance, CA, USA) and a Polar C_18_ Security Guard cartridge (Phenomenex) of 4 x 3.0 mm. The mobile phases consisted of water + formic acid 0.1 % v/v (eluent A) and acetonitrile (eluent B). The gradient elution was: 10-25% B in 0-25 min, 25% B in 25-30 min, 25-50% B in 30-40 min, 50-100% B in 40-42 min, 100% in 42-47 min. Then, eluent B was returned to 10% with a 7 min stabilization time. The flow rate was 0.4 ml min^-1^.

The HPLC system was connected to an ion trap mass spectrometer (Esquire 6000, Bruker Daltonics, Billerica, MA, USA) equipped with an electrospray interface. The scan range was at m/z 100–1200 with a speed of 13,000 Da/s. The ESI conditions were: drying gas (N_2_) flow rate and temperature, 10 mL/min and 365 ºC; nebulizer gas (N_2_) pressure, 50 psi; capillary voltage, 4500 V; capillary exit voltage, -117.3 V. We used the auto MS^n^ mode for the acquisition of MS^n^ data, with isolation width of 4.0 m/z, and fragmentation amplitude of 0.6 V (MS^n^ up to MS^4^).

**File S3: Assays for Total Phenolic and Flavonoid Contents**

The total phenolic content was determined by employing the methods given in the literature with some modiﬁcation. Sample solution (0.25 mL) was mixed with diluted Folin–Ciocalteu reagent (1 mL, 1:9, v/v) and shaken vigorously. After 3 min, Na_2_CO_3_ solution (0.75 mL, 1%) was added and the sample absorbance was read at 760 nm after 2 h incubation at room temperature. The total phenolic content was expressed as milligrams of gallic acid equivalents (mg GAE/g extract) [1].

The total ﬂavonoid content was determined using the AlCl_3_ method. Brieﬂy, sample solution (1 mL) was mixed with the same volume of aluminum trichloride (2%) in methanol. Similarly, a blank was prepared by adding sample solution (1 mL) to methanol (1 mL) without AlCl_3_. The sample and blank absorbances were read at 415 nm after a 10 min incubation at room temperature. The absorbance of the blank was subtracted from that of the sample. Rutin was used as a reference standard and the total ﬂavonoid content was expressed as milligrams of rutin equivalents (mg RE/g extract) [1].

**File S4: Determination of Antioxidant and Enzyme Inhibitory Effects**

Antioxidant (DPPH and ABTS radical scavenging), reducing power (CUPRAC and FRAP), phosphomolybdenum and metal chelating (ferrozine method) and enzyme inhibitory activities (cholinesterase (Elmann’s method), tyrosinase (dopachrome method), α-amylase (iodine/potassium iodide method) and α-glucosidase (chromogenic PNPG method)) were determined using the methods previously described [1,2].

For the DPPH (2,2-diphenyl-1-picrylhydrazyl) radical scavenging assay: Sample solution was added to 4 mL of a 0.004% methanol solution of DPPH. The sample absorbance was read at 517 nm after 30 min incubation at room temperature in the dark. DPPH radical scavenging activity was expressed as milligrams of trolox equivalents (mg TE/g extract).

For ABTS (2,2′-azino-bis(3-ethylbenzothiazoline)-6-sulfonic acid) radical scavenging assay: Brieﬂy, ABTS^+^ was produced directly by reacting 7 mM ABTS solution with 2.45 mM potassium persulfate and allowing the mixture to stand for 12–16 in the dark at room temperature. Prior to beginning the assay, ABTS solution was diluted with methanol to an absorbance of 0.700 ± 0.02 at 734 nm. Sample solution was added to ABTS solution (2 mL) and mixed. The sample absorbance was read at 734 nm after 30 min incubation at room temperature. The ABTS radical scavenging activity was expressed as milligrams of trolox equivalents (mg TE/g extract).

For CUPRAC (cupric ion reducing activity) activity assay: Sample solution was added to premixed reaction mixture containing CuCl_2_ (1 mL, 10 mM), neocuproine (1 mL, 7.5 mM) and NH_4_Ac buffer (1 mL, 1 M, pH 7.0). Similarly, a blank was prepared by adding sample solution (0.5 mL) to premixed reaction mixture (3 mL) without CuCl_2_. Then, the sample and blank absorbances were read at 450 nm after 30 min incubation at room temperature. The absorbance of the blank was subtracted from that of the sample. CUPRAC activity was expressed as milligrams of trolox equivalents (mg TE/g extract).

For FRAP (ferric reducing antioxidant power) activity assay: Sample solution was added to premixed FRAP reagent (2 mL) containing acetate buffer (0.3 M, pH 3.6), 2,4,6-tris(2-pyridyl)-S-triazine (TPTZ) (10 mM) in 40 mM HCl and ferric chloride (20 mM) in a ratio of 10:1:1 (v/v/v). Then, the sample absorbance was read at 593 nm after 30 min incubation at room temperature. FRAP activity was expressed as milligrams of trolox equivalents (mg TE/g extract).

For phosphomolybdenum method: Sample solution was combined with 3 mL of reagent solution (0.6 M sulfuric acid, 28 mM sodium phosphate and 4 mM ammonium molybdate). The sample absorbance was read at 695 nm after 90 min incubation at 95°C. The total antioxidant capacity was expressed as millimoles of trolox equivalents (mmol TE/g extract).

For metal chelating activity assay: Brieﬂy, sample solution was added to FeCl_2_ solution (0.05 mL, 2 mM). The reaction was initiated by the addition of 5 mM ferrozine (0.2 mL). Similarly, a blank was prepared by adding sample solution (2 mL) to FeCl_2_ solution (0.05 mL, 2 mM) and water (0.2 mL) without ferrozine. Then, the sample and blank absorbances were read at 562 nm after 10 min incubation at room temperature. The absorbance of the blank was subtracted from that of the sample. The metal chelating activity was expressed as milligrams of EDTA (disodium edetate) equivalents (mg EDTAE/g extract).

For cholinesterase (ChE) inhibitory activity assay: Sample solution was mixed with DTNB (5,5-dithio-bis(2-nitrobenzoic) acid (125 µL) and AChE (acetylcholinesterase (Electric ell acetylcholinesterase, Type-VI-S, EC 3.1.1.7)), or BChE (butyrylcholinesterase (horse serum butyrylcholinesterase, EC 3.1.1.8)) solution (25 μL) in Tris–HCl buffer (pH 8.0) in a 96-well microplate and incubated for 15 min at 25 °C. The reaction was then initiated with the addition of acetylthiocholine iodide (ATCI) or butyrylthiocholine chloride (BTCl) (25 μL). Similarly, a blank was prepared by adding sample solution to all reaction reagents without enzyme (AChE or BChE) solution. The sample and blank absorbances were read at 405 nm after 10 min incubation at 25 °C. The absorbance of the blank was subtracted from that of the sample and the cholinesterase inhibitory activity was expressed as milligrams of galanthamine equivalents (mg GALAE/g extract).

For tyrosinase inhibitory activity assay: Sample solution was mixed with tyrosinase solution (40 μL) and phosphate buffer (100 μL, pH 6.8) in a 96-well microplate and incubated for 15 min at 25 °C. The reaction was then initiated with the addition of L-DOPA (40 μL). Similarly, a blank was prepared by adding sample solution to all reaction reagents without enzyme (tyrosinase) solution. The sample and blank absorbances were read at 492 nm after 10 min incubation at 25 °C. The absorbance of the blank was subtracted from that of the sample and the tyrosinase inhibitory activity was expressed as milligrams of kojic acid equivalents (mg KAE/g extract).

For α-amylase inhibitory activity assay: Sample solution was mixed with α-amylase solution (ex-porcine pancreas, EC 3.2.1.1) (50 μL) in phosphate buffer (pH 6.9 with 6 mM sodium chloride) in a 96-well microplate and incubated for 10 min at 37 °C. After pre-incubation, the reaction was initiated with the addition of starch solution (50 μL, 0.05%). Similarly, a blank was prepared by adding sample solution to all reaction reagents without enzyme (α-amylase) solution. The reaction mixture was incubated 10 min at 37 °C. The reaction was then stopped with the addition of HCl (25 μL, 1 M). This was followed by addition of the iodine-potassium iodide solution (100 μL). The sample and blank absorbances were read at 630 nm. The absorbance of the blank was subtracted from that of the sample and the α-amylase inhibitory activity was expressed as millimoles of acarbose equivalents (mmol ACAE/g extract).

For α-glucosidase inhibitory activity assay: Sample solution was mixed with glutathione (50 µL), α-glucosidase solution (from Saccharomyces cerevisiae, EC 3.2.1.20) (50 µL) in phosphate buffer (pH 6.8) and PNPG (4-N-trophenyl-α-D-glucopyranoside) (50 µL) in a 96-well microplate and incubated for 15 min at 37 °C. Similarly, a blank was prepared by adding sample solution to all reaction reagents without enzyme (α-glucosidase) solution. The reaction was then stopped with the addition of sodium carbonate (50 µL, 0.2 M). The sample and blank absorbances were read at 400 nm. The absorbance of the blank was subtracted from that of the sample and the α-glucosidase inhibitory activity was expressed as millimoles of acarbose equivalents (mmol ACAE/g extract).

**References**

1. Uysal, S.; Zengin, G.; Locatelli, M.; Bahadori, M.B.; Mocan, A.; Bellagamba, G.; De Luca, E.; Mollica, A.; Aktumsek, A. Cytotoxic and enzyme inhibitory potential of two *Potentilla* species (*P. speciosa* L. and *P. reptans* Willd.) and their chemical composition. *Front. Pharmacol.* **2017**, *8*: 290.

2. Grochowski, D.M.; Uysal, S.; Aktumsek, A.; Granica, S.; Zengin, G.; Ceylan, R.; Locatelli, M.; Tomczyk, M. *In vitro* enzyme inhibitory properties, antioxidant activities, and phytochemical profile of *Potentilla thuringiaca*. *Phytochem. Lett.* **2017**, *20*: 365–372.

TABLE S1: Total phenolic and flavonoid contents of the extracts of *S.* *enneaphylla* (SE), *S. pulcherrima* (SP) and *S. saetabensis* (SS), each one from two different locations, extracted in methanol and water.

| **Samples** | | | **Total phenolic content (mg GAE/g)** | **Total flavonoid content (mg RE/g)** |
| --- | --- | --- | --- | --- |
| ***S. enneaphylla*** | **MeOH** | **SE1** | 61.2 ± 0.6^a^ | 43 ± 4^e^ |
|  |  | **SE2** | 40 ± 1^d^ | 89.2 ± 0.9^a^ |
|  | **H_2_O** | **SE1** | 21 ± 1^g^ | 5.8 ± 0.3^gh^ |
|  |  | **SE2** | 19.39 ± 0.07^gh^ | 9.4 ± 0.6^fg^ |
|  |  |  |  |  |
| ***S. pulcherrima*** | **MeOH** | **SP1** | 43.3 ± 0.8^c^ | 65 ± 2^d^ |
|  |  | **SP2** | 43.8 ± 0.4^c^ | 66.3 ± 1.4^cd^ |
|  | **H_2_O** | **SP1** | 31.2 ± 0.1^e^ | 11.1 ± 0.5^f^ |
|  |  | **SP2** | 17.8 ± 0.2^h^ | 5.4 ± 0.6^gh^ |
|  |  | |  |  |
| ***S. saetabensis*** | **MeOH** | **SS1** | 50.6 ± 0.4^b^ | 70.0 ± 0.6^c^ |
|  |  | **SS2** | 40.0 ± 0.7^d^ | 77 ± 1^b^ |
|  | **H_2_O** | **SS1** | 19.2 ± 0.2^gh^ | 4.8 ± 0.2^h^ |
|  |  | **SS2** | 27.3 ± 0.8^f^ | 8.5 ± 0.6^fgh^ |

Values expressed are means ± S.D. of three parallel measurements. GAE: Gallic acid equivalent; RE: Rutin equivalent. Different letters indicate significant differences in the extracts (*p*<0.05).

FIGURE S1: HPLC-ESI/MS^n^ base peak chromatograms (BPC) of the aqueous extracts of (A) *S. enneaphylla* (SE1), (B) *S*. *pulcherrima* (SP1), and (C) *S. saetabensis* (SS2).

FIGURE S2: Ranges of flavonoids, phenolic acids and TIPC values for the three *Sarcocapnos* species.


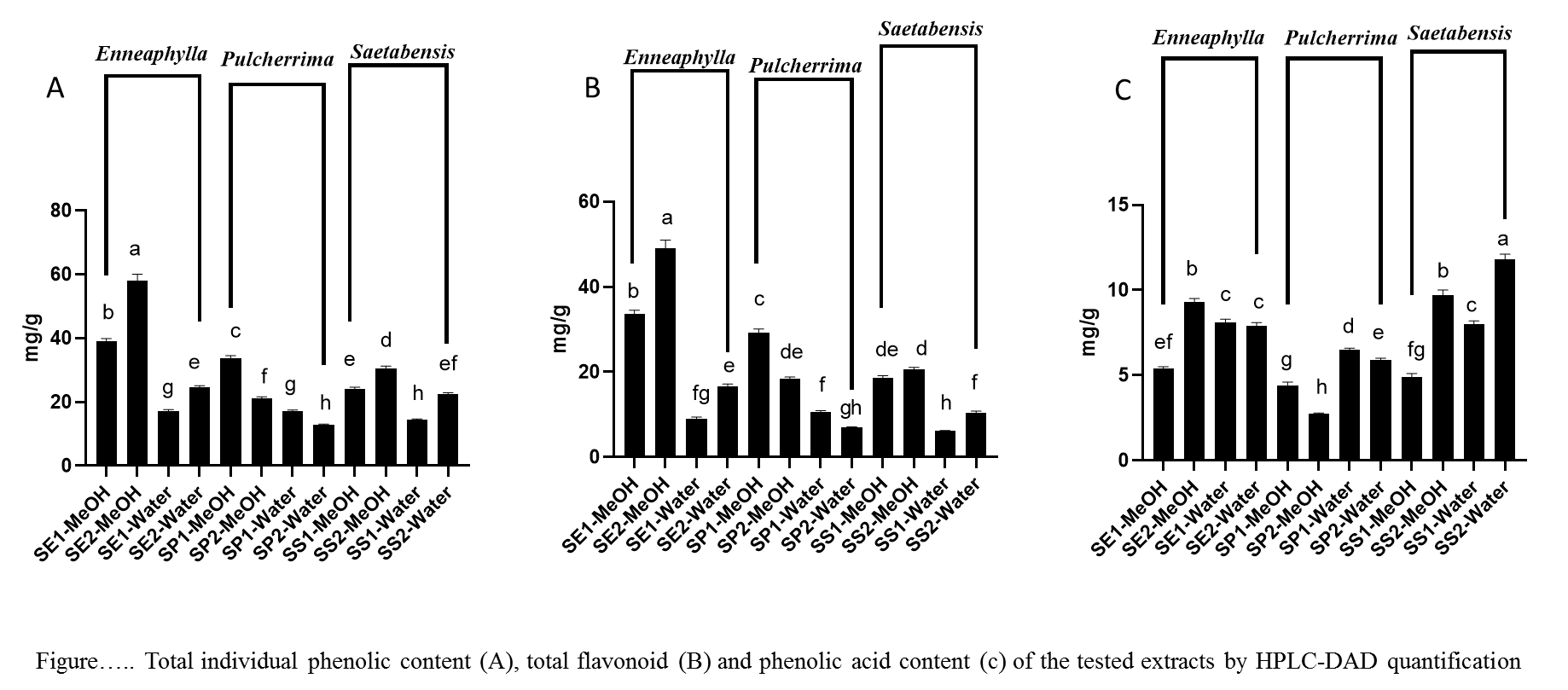


FIGURE S3: Total individual phenolic content (A), total flavonoid content (B) and phenolic acid content (C) of the extracts by HPLC-DAD quantification. Different letters indicate significant differences in the extracts (p<0.05).
